# Strain-dependent motility defects and suppression by a *flhO* mutation for *B. subtilis* bactofilins

**DOI:** 10.1186/s13104-022-06048-6

**Published:** 2022-05-13

**Authors:** Sven Holtrup, Peter L. Graumann

**Affiliations:** 1 SYNMIKRO, Zentrum Für Synthetische Mikrobiologie, Karl-von-Frisch-Str. 14, 35043 Marburg, Germany; 2 Fachbereich Chemie, Hans-Meerwein-Straße 4, 35032 Marburg, Germany

**Keywords:** *Bacillus subtilis*, Bactofilins, Biofilm formation, Flagellum, Motility, Swarming

## Abstract

**Objective:**

Bactofilins can assemble into polymeric structures and play important roles in cell shape maintenance, chromosome segregation and motility. *Bacillus subtilis* bactofilins BacE and BacF were shown to be important for swimming motility in strain PY79, and single gene deletions were reported to be lethal, in contrast to a double *bacEF* deletion.

**Results:**

Extending this work, we show that motility defects vary between different *B. subtilis* strains, with strain 168 showing no defect in motility, and 3610 showing delayed induction of swimming. Generation of single gene deletions in PY79 was possible by transferring corresponding deletions from 168. In the natural isolate 3610, gene deletions also showed a negative effect on biofilm formation, revealing an additional function for BacE and BacF. A spontaneous arising suppressor mutation in PY79 was mapped to the *flhO* gene, a constituent of the flagellum, which obtained an 18 amino acid extension at its C-terminus. Our findings show that bactofilin gene deletions lead to different motility phenotypes dependent on the strain background, and affect biofilm formation in the natural isolate 3610. Our data reinforce the idea of a connection between bactofilins and motion via the flagellum, and suggest that they operate in a switch like manner.

**Supplementary Information:**

The online version contains supplementary material available at 10.1186/s13104-022-06048-6.

## Introduction

Bactofilins are a widespread class of small filament-forming proteins. Homologs have been found all around the bacterial phylogenetic tree and are frequently duplicated within genomes [1, 2]. Additionally, they were recently found in some eukaryotic and archaeal species [2]. Bactofilins are defined by a central (right-handed) β-helical bactofilin domain (DUF583) necessary for polymerization, which is usually neighbored by short unstructured N- and C- terminal domains of various lengths that potentially confer functional specificity [1, 3]. Co-factor independent polymerization into highly organized filament or patch-like structures was demonstrated in vitro [2] and polymers were found to be resistant against a wide range of extreme conditions [1, 4]. The cryo-structure of the *Thermus thermophilus* bactofilin TtBacA recently suggested a head-head polymerization mechanism and TtBacA was also found to exhibit intrinsic membrane affinity [2].

Among the best studied examples are BacA from *Caulobacter crescentus* that is essential for stalk formation [2], and *Myxococcus xanthus* bactofilins BacNOP that were shown to interact with the chromosome segregation machinery [5]. Another well-studied example represents CcmA from *Helicobacterpylori,* which acts in line with a dynamic peptidoglycan (PG)-modifying machinery that locally changes the cross-linking pattern in the PG-sacculus and thus enables the typical cork-screw like shape of the bacterium [6].

The gram-positive model organism *B. subtilis* possesses two bactofilin homologs, BacE (encoded by *bacE*, earlier named *yhbE*) and BacF (*yhbF*) [7]. Using fluoresce-microscopy it was shown that both proteins localize as discrete 60–70 nm assemblies at the cell membrane with BacF assemblies being relatively static and partially co-localizing with the flagella basal-bodies and BacE structures being fewer per cell and highly mobile. Interestingly, the exchange of *bacEF* by a tetracycline resistance cassette resulted in loss of swimming motility in PY79. Further investigation unveiled that the cells were not able to generate neither the flagellar hook nor the filament [7].

Flagella-mediated motility is a life-saving feature for many bacteria, enabling them to survive in environments that are dominated by nutrient gradients. Due to its drastic influence on the lifestyle of *B. subtilis*, assembly of functional flagella is a time and energy costly process that involves over 20 structural proteins and underlies a complex assembly-order. It is still unclear how bactofilins may act on the formation of the flagellum, or the regulation thereof. *B. subtilis* lab strains exhibit two forms of active, flagella driven movement, swimming and swarming [8, 9]. Swimming refers to individual cells moving in three-dimensional space through liquid media. In swarming on the other hand, cells form groups to join their flagella forces to move in two dimensional space over solid media [10].

All attempts of creating *bacE* or *bacF* single knock-out strains failed in our earlier study. Both genes could only be deleted in the presence of an ectopic copy of both genes, suggesting that the loss of single genes is lethal [7]. To our surprise, we found single *bacE* and *bacF* mutants in the *Bacillus-*genetic-stock-center (BGSC, Columbus, Ohio) deletion collection [11] (strain 168). To clarify if strain-dependent differences exist, we investigated effects of single or double bactofilin deletions in three strains, laboratory strains PY79 and 168, and the undomesticated strain NCIB 3610 carrying *comI*^*Q12L*^ mutation [12]. We found motility effects to very different extents in the three strains, and an additional defect in biofilm formation in strain 3610. Interestingly, *bacF*-mutant colonies in strain PY79 frequently reverted to swarming cells after extended cultivation on solid media. Complementing this work, we created a suppressor mutant of PY79 *bacEF::tet* that is as motile as its wildtype progenitor and found a single nucleotide deletion in the putative rod-gene *flhO* as causative reason. This work clarifies and extends results found in El Andari et al. [7]. Results presented here were generated within the duration of around one year.

## Main text

### Results

#### Single and double bacEF mutants cause different motility defects in different strain backgrounds

As reported earlier [7], a double *yhbEF* (from now on called *bacEF*) deletion resulted in cells of the lab-strain PY79 lacking filaments and hooks, and the construction of single bactofilin knock-out mutants failed. Motility in the *bacEF* double mutant strain could not be complemented by ectopically expressing either of these genes, only the expression of both proteins was able to restore motility. However, within the *B. subtilis* mutant strain collection [11], we found single knock-out strains of these genes, albeit in the background of the other commonly utilized lab-strain 168. By using total DNA isolated from this strain (also referred to as genomic DNA or gDNA) for transformation, we were able to obtain single knock-outs also in PY79. These experiments show that lethality of the double deletion was an artefact of the DNA construct that was chosen to create the deletion of both genes, which had been analogous to constructs for single gene deletions. We next created a *bacEF* double mutant in 168 by using genomic-DNA from PY79 *bacEF*::*tet,* and analogously, single and double gene deletion strains in the undomesticated strain 3610 (strains are listed in Table 1).

**Fig. 1:**
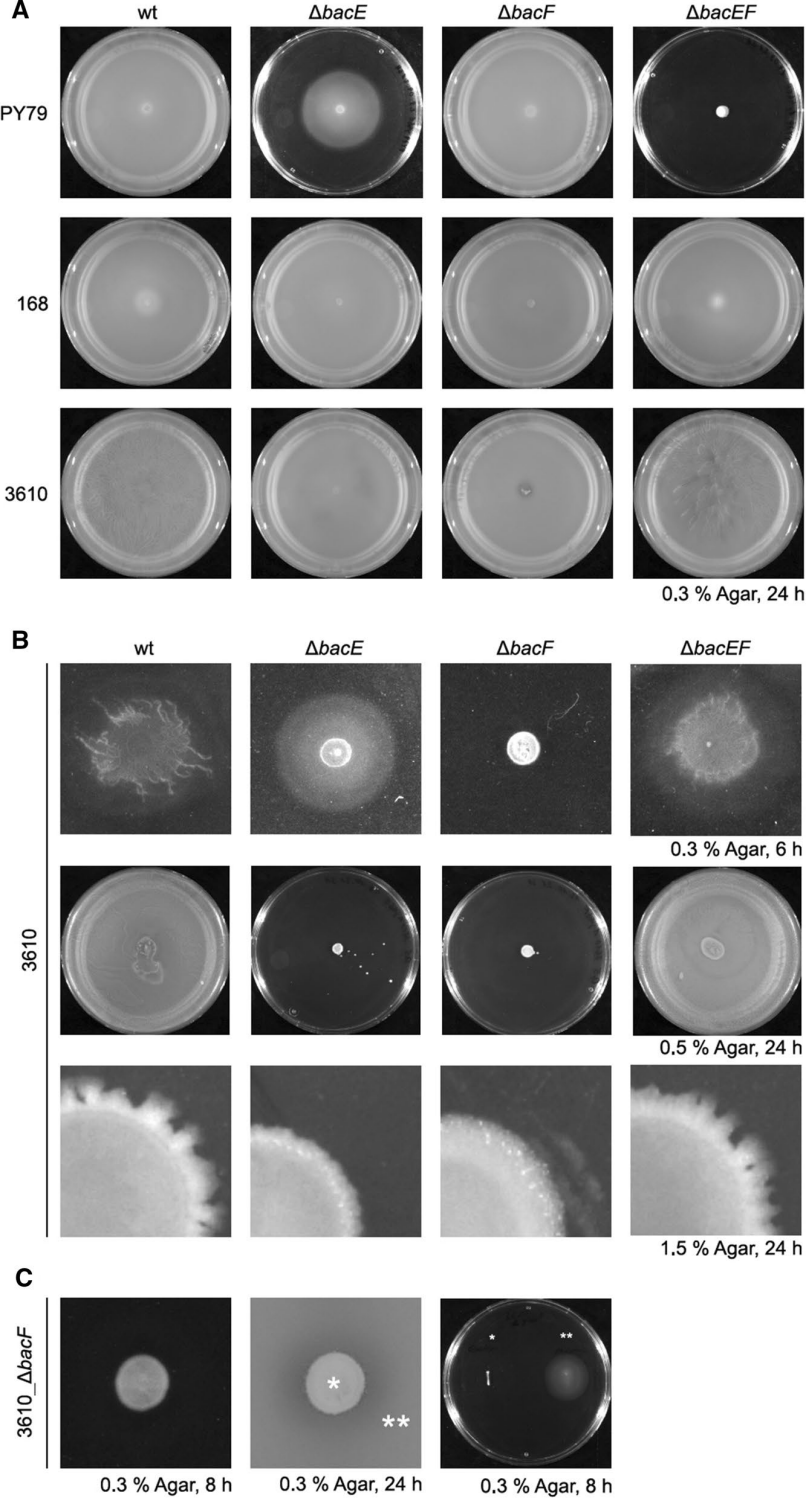
Motility of bactofilin knock-out mutants** A** Flagella mediated motility assayed on 0.3% LB-agar plates imaged after 24 h incubation at 30°C. **B** Colony growth of 3610 bactofilin mutants on 0.3% agar plates (upper-lane) after 6 h of incubation, 0.5% agar plates after 24 h (middle lane) incubation and on 1.5% agar after 24 h of incubation (third lane). **C** Same colony of 3610_Δ*bacF* on 0.3% agar after 6 h and 24 h incubation. Colonies frequently developed motility suppressor mutations between 8 and 24 h post inoculation. Cells from the center (indicated by a single asterisk) and the periphery (double asterisk) were comparatively streaked on a new 0.3% agar plate and incubated for 8 h

All strains were verified by PCR and were assayed on 0.3% soft agar plates (Fig. 1 A) (primer sequences are given in Additional file 1). As reported earlier, surface movement under these conditions is due to flagella-based motility. In agreement with our earlier study, *bacE* mutant cells showed a motility defect in strain PY79, while *bacF* mutants were able to spread over the surface similar to wild type cells. Curiously, double mutant cells lacked any detectable motility in strain PY79 (Fig. 1A). Interestingly, in strain 168, neither the single nor the double deletions resulted in a loss of motility (Fig. 1A). These results show that PY79 cells show strikingly different requirements for motility than 168 cells. In the background of 3610, we did not observe any effect of single or double deletions on motility 24 h after incubation (Fig. 1A), suggesting that defects found in PY79 are laboratory artefacts. However, 6 h after incubation, 3610 *bacF* mutant cells did not show any spreading across the surface, in contrast to 3610 wild type cells, which showed spreading including visible threads of cells showing high motility (Fig. 1B). Also, *bacE* mutant cells showed a phenotype of apparent swimming activity, but these cells lacked structured thread-like structures (Fig. 1B). Surprisingly, *bacEF* double mutant cells showed wild type-like behavior, showing that a deletion of both genes suppresses single deletion phenotypes. These findings show that bactofilin deletions do impact motility in natural isolate *B. subtilis* cells, but seemingly in a switch-like manner rather than in a structural manner, because induction of swarming appears to be delayed, rather than swimming disturbed per se.

As an extension of these analyses, we found that a majority of *bacF* mutant cells of strain 3610 developed suppressor mutations occurring as early as 8 to 24 h (Fig. 1C), much earlier than we observed for similar mutant cells of strain PY79 (not shown). While cells in the center of the colony continued to lack motility after restreaking (Fig. 1C, right panel), cells from the outer areas continued to swim. We were curious to investigate the nature of such regaining of motility (see further below).

#### Bactofilin deletions influence biofilm formation

In addition to a delay in swimming activity, we found that single bactofilin gene deletions in strain 3610 were not able to form biofilms on soft agar (0.3%), unlike wild type cells, which formed structured colonies (Fig. 1B). Different from its domesticated derivatives PY79 and 168, strain 3610 creates a thick biofilm when grown on solid medium, including many differently differentiated cells and accompanied by the secretion of a complex matrix [13]. Therefore, defects in biofilm formation are undetectable in laboratory strains. At the transition to stationary phase, *B. subtilis* cells induce the secretion of lipopeptide surfactin, which reduces surface tension (amongst other functions), allowing cells to use flagellar motion to move on semi-solid agar of 0.5%. This so-called swarming motion allows for spreading also on higher concentrated agar plates as shown in Fig. 1B, but not on 1.5% agar plates. In contrast to wild-type 3610 cells, single *bacE* or *bacF* mutant cells did not show any swarming motility (Fig. 1B), while a double *bacEF* deletion restored swarming motility as well as biofilm formation (Fig. 1B). In agreement with single deletions affecting spreading on (semi)-solid agar plates, colonies of *bacE* or *bacF* mutant cells showed a rather smooth outer edge, while that of wild type or double mutant cells was frayed and ragged (Fig. 1B). Thus, while BacE and BacF are important for motility in the absence of a water layer on the surface in natural isolate *B. subtilis* cells, a requirement that is suppressed by the lack of both of them, they are not required for motility on soft agar, i.e. in the presence of a water layer.

*Sfp* encodes for a 4-phosphopantetheinyl transferase, required for the production of surfactin that facilitates swarming by reduction of surface tension. SwrAA is a regulator of flagellar synthesis and enhances *sigD*-controlled transcription of the large flagellar operon. Both defects are related to loss of swarming and multicellularity in laboratory strains [14]. It will be interesting to find out at which level bactofilins affect swarming activity, but clearly, care must be taken in how far strain backgrounds influence the effects of bactofilin gene deletions.

#### PY79 bactofilin motility defects can be suppressed by an extension of the flhO open reading frame

**Fig. 2:**
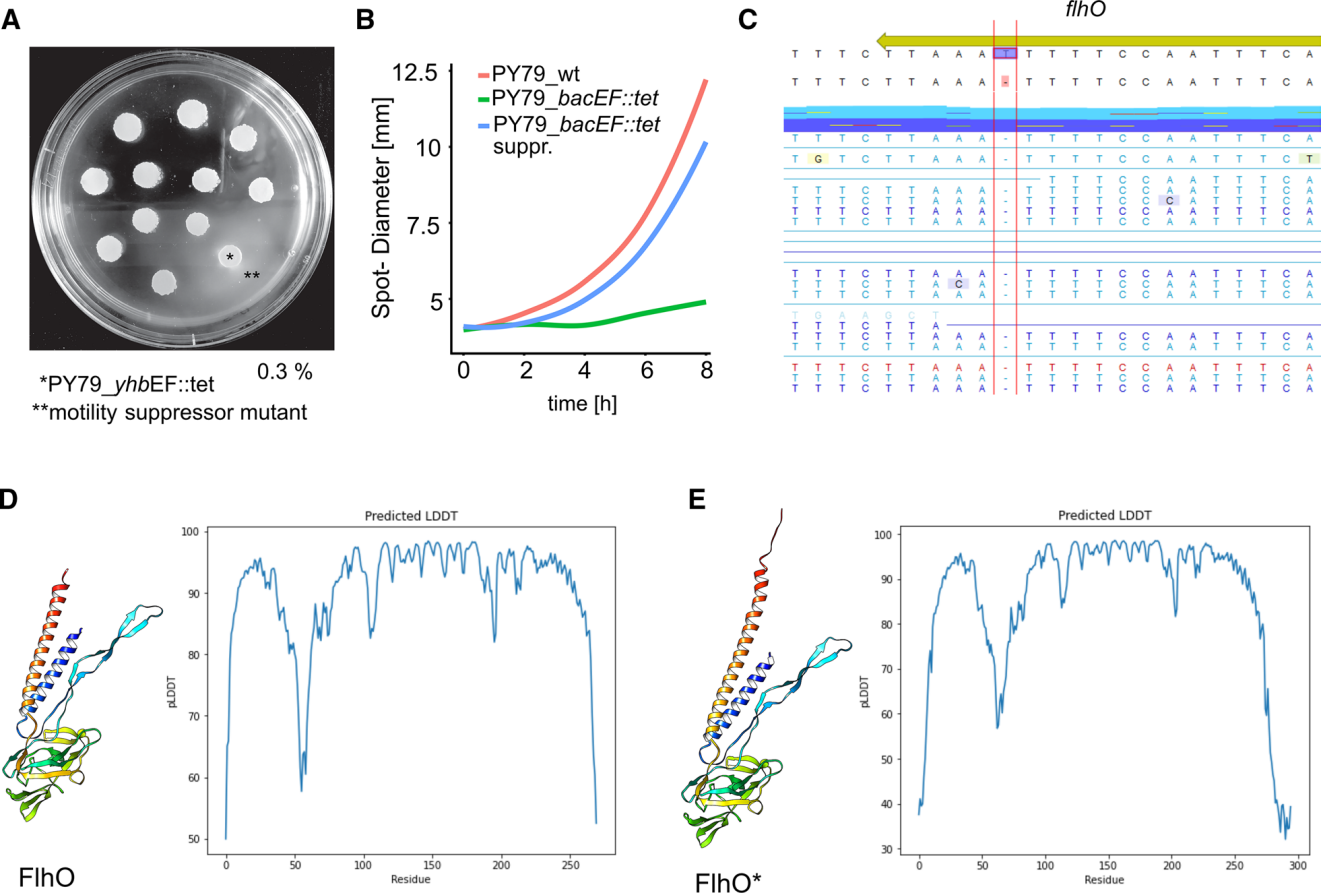
**A** Generation of a PY79 *bacEF::tet* suppressor mutant. Mutants (double asterisk) swarm out from their mother colony (single asterisk). **B** Colony diameter growth on 0.3% soft agar over 8 h at 30 °C. Mutants regained motility comparable to that of the wt.** C** Genome sequencing unveiled a single nucleotide deletion in the gene *flhO,* causing a frameshift. The new ORF encodes for a FlhO with the C-terminal extension "FKKTEEKNGGHQLCSGQC".** D** Model of the native *B. subtilis* FlhO and its C-terminally expanded counterpart, FlhO* (**E**), as generated by the alphafold algorithm [15] (AlphaFold v2.1.0, Creative Commons Attribution-NonCommercial 4.0 International license, CC BY-NC 4.0) in Colab (Apache 2.0 license). Predicted local superposition-free scores (LDDT) are illustrated for FlhO and FlhO*-residues respectively. Molecular graphics were generated with UCSF Chimera (developed by the Resource for Biocomputing, Visualization, and Informatics at the University of California, San Francisco, with support from NIH P41-GM103311), peptide chains are colored blue to red from N- to C-terminus by the rainbow colors

In order to gain further insight into the mechanisms underlying the flagellar defect of a bactofilin double deletion of PY79, we generated suppressor mutant colonies. To this end, we dropped multiple spots onto 0.3% soft agar plates and incubated them for several days (note the difference to the single *bacF* deletion in 3610, Fig. 1C) until we observed motile cells that were swarming out from one of the spots (Fig. 2 A). We compared surface swarming of the suppressor mutant to the PY79 double mutant strain and the PY79 wild type by measuring spot diameter over an 8 h duration. As illustrated in Fig. 2B, mutant cells that had regained motility exhibited surface spreading comparable to that of wild type cells. Both strains, PY79 *bacEF::tet*^*sup*^ and its motile derivative, were sent for whole-genome sequencing. Comparing both datasets, we found a single point mutation within the reading frame of the flagellar rod protein FlhO, where a thymidine close to the stop codon had been deleted*,* resulting in a frameshift. There was no other mutation in the genome of the suppressor strain, clearly identifying the mutation as the relevant change for regaining of motility. The new ORF of *flhO* encodes for an 18 aa extension at its C-terminus (FKKTEEKNGGHQLCSGQC). We used the alpha-fold algorithm [15] for modeling the native *B. subtilis* FlhO structure (Fig. 2 D) and its extended counterpart, labeled FlhO* (Fig. 2 E). The predicted local superposition-free score (pLDDT) suggest a good confidence for FlhO* except for a small area around residue 60 where the pLDDT is only at around 60 and the extended C-terminal sequence that gives a low confidence. As a consequence, the peptide sequence added to the C-terminal FlhO alpha-helix is predicted as unstructured, but does not appear to change the overall fold of FlhO. It is unclear, how the FlhO extension can compensate for the lack of bactofilins.

FlhO is a structural protein in the Rod-complex of the flagellum, and cells lacking FlhO are deficient in synthesizing a filament. FlhO*,* together with FlhP is encoded outside of the 27 kb *che/fla* operon under the control of the SigD-dependend *flhO-*promotor. Assembly of the *B. subtilis* rod-complex follows a distinct assembly order, starting from the distal FliE over FlgB, FlgC and FlhO to the peripheral FlhP. Intermediates were found to be metastable or subject to proteolysis [16]. Because bactofilin mutants have been shown to be deficient in the hook structure, arising from the rod complex, our finding of FlhO acting as a suppressor mutation represents another hint on the role of *Bacillus* bactofilins on flagella assembly. Evidently, the requirement for bactofilin activity is different for different *B. subtilis* strains, and our finding that single bactofilin deletions can lead to motility defects, while double deletions in strain 3610 show no defects suggests that bactofilins play a regulatory role and possible set up a negative feed-back loop (Table 1).

**Table 1:** List of strains used in this study

Strain	Description	Resistance-marker	References
PY79 (wild type)	*B. subtilis* lab-strain PY79	–	[17]
PY79 bacEF::tet	*bacEF* replaced by tetracycline-resistance cassette	Tetracycline	[7]
PY79 bacEF::tet _mot. Suppr	suppressor mutant, generated from PY79 bacEF::tet [7]	Tetracycline	This study
PY79 ΔbacE	Δ*bacE*, generated using gDNA from 168 ΔbacE [12]	Kanamycin	This study
PY79 ΔbacF	Δ*bacF*, generated using gDNA from 168 ΔbacF [12]	Kanamycin	This study
168 (wild type)	*B. subtilis* lab-strain 168	–	[18]
168 ΔbacE	Δ*bacE*-strain	Kanamycin	[11]
168 ΔbacF	Δ*bacF*-strain	Kanamycin	[11]
168 bacEF::tet	*ΔbacEF* strain, generated by using gDNA from PY79 bacEF::tet [7]	Tetracycline	This study
3610 (wild type)	NCBI_3610, comIQ12L	-	[12]
3610 ΔbacE	Δ*bacE* strain, generated using gDNA from 168 ΔbacE	Kanamycin	This study
3610 ΔbacF	Δ*bacF* strain, generated using gDNA from 168 ΔbacF	Kanamycin	This study
3610 bacEF::tet	*ΔbacEF* strain, generated by using gDNA from PY79 bacEF::tet	Tetracycline	This study

## Limitations

The identification of a single suppressor (flhO*) for a *bacEF* deletion in PY79 does not mean that there could be other mutations, which might compensate for the lack of BacF. These could also appear in structures other than the flagellum. Delay of swimming activity seen in *bacF* mutant cells in strain 3610, and arising of suppressors is highly stochastic, so this effect is highly variable and difficult to (Additional file 1) quantify, also the number of colonies developing suppressor mutations.

## Supplementary Information

**Additional file1 Table S1:** List of primers used in this study

## Data Availability

All data generated or analysed during this study are included in this published article. Raw images are available upon request at the corresponding author.

## Abbreviations

PG: Peptidoglycan
LDDT: Local superposition-free score
Wt: Wild type
BGSC: Bacillus Genetic Stock Centre
gDNA: Genomic DNA
