# Supplementary material for: Strain-dependent motility defects and suppression by a flhO mutation for B. subtilis bactofilins
Source: BMC Res Notes. 2022 May 13;15:168. doi: 10.1186/s13104-022-06048-6 (PMC9103452; doi:10.1186/s13104-022-06048-6)
Supplement: Supplementary file 1 — Additional file1 Table S1: List of primers used in this study [file 13104_2022_6048_MOESM1_ESM.docx]

**Materials and Methods**

**Bacterial growth conditions**

*B. subtilis* strains were grown on LB-Agar (Carl Roth, Germany) plates supplemented with Kan/Tet if needed and incubated at 30°C. If not denoted differently, for liquid cultures 3,5 ml LB (Carl Roth, Germany) supplemented with respective antibiotics was inoculated directly from cryo-stocks.

**Strain construction**

All gene knock-out strains were generated by natural competence based on genomic DNA of *B. subtilis*. 10 ml competence media were prepared from 1 ml 10xMC (14.04% (w/v) K_2_HPO_4_, 5.24% (w/v) KH_2_PO_4_, 20% (w/v) glucose), 0.33 ml MgSO_4_ (1 M) and 8.7 ml A. dest. and inoculated with overgrown *B. subtilis* culture to an OD_600_ between 0.08-0.1. The culture was incubated for around 3 h at 37°C, 200 rpm until they reached OD_600_ 1.5. Subsequently, 5-10 µg of genomic DNA were added to 1 ml aliquots of the culture and incubated for 2 h at 37°C, 200 rpm before plating onto selective media. All strains *B. subtilis* strains are listed in Table 1.

**Isolation of gDNA from *B. subtilis***

Total DNA was isolated from *B. subtilis* cells using the *innuPREP* Bacteria DNA kit by Jena-Analytics (Germany), according to the manufacturer's instructions.

**Motility assay**

Motility of strains was assayed on soft-agar. Plates containing 25 ml softagar (Bacto agar dissolved in LB-media, percentages are given in weight per volume) were prepared the on the previous day and dried around 20 min prior to the experiment. 5 µl drops of overgrown over-night culture were spotted onto the agar. Plates were incubated at 30°C and documented every two hours.

**Image analysis and visualization**

Plates for analysis were imaged using the BioRad Chemi-Doc documentation system. Image processing was done using Fiji and data analysis and visualization was done in R-Studio using the packages dplyr and ggplot2 [1]. FlhO models were generated by using [AlphaFold v2.1.0](https://doi.org/10.1038/s41586-021-03819-2) [2] (Creative Commons Attribution-NonCommercial 4.0 International license, CC BY-NC 4.0) in Colab (Apache 2.0 license).

**Supplementary Discussion**

Knowledge on cellular and environmental factors that initiate the planktonic/sessile lifestyle transformation is still incomplete. The process involves two sensor histidin-kinases KinC and KinD [3] and the response-regulator Spo0A, a master-regulator of cell-differentiation in *B. subtilis* [4]. When a threshold-level of phosphorylated Spo0A is reached, the expression of *sinI gets* upregulated. The gene product SinI antagonizes the repressor SinR and thereby enables transcription of biofilm genes (*eps-O, tapA-sipW-tasA, bsiA*) [5, 6]. Additionally, a suppressor screen on *sinR* mutants discovered the DNA-binding proteins RemA and RemB as activators of the three major biofilm operons [7]. Interestingly, expression of *bacE* and *bacF* has been found upregulated when cells were grown on solid media [8], indicating that they could also play a role in this adaption. BacF appears to have a bigger impact on motility, because the knock-out mutant strain does not exhibit surface spreading. Additionally, BacF was shown to localize more statically to the basal bodies as compared to BacE [9].

**Table S1:** List of primers used in this study

| Primer name | Sequence | Reference |
| --- | --- | --- |
| bacE_ApaI_for | catgggcccagatacatggagacgttgaa | [9] |
| bacE_EcoRI_rev | catgaattccaactttgtggatgtttcaac | [9] |
| bacF_ApaI_for | catgggcccaaagtgaatgtaaaggcgatc | [9] |
| bacF_EcoRI_rev | catgaattccagttttgttttttctttaatct | [9] |

**References**

1. Wickham H, Averick M, Bryan J, Chang W, McGowan LDA, François R, Grolemund G, Hayes A, Henry L, Hester J: **Welcome to the Tidyverse**. *Journal of open source software* 2019, **4**(43):1686.

2. Jumper J, Evans R, Pritzel A, Green T, Figurnov M, Ronneberger O, Tunyasuvunakool K, Bates R, Žı́dek A, Potapenko A *et al*: **Highly accurate protein structure prediction with AlphaFold**. *Nature* 2021, **596**(7873):583–589-583–589.

3. Chen Y, Cao S, Chai Y, Clardy J, Kolter R, Guo J-h, Losick R: **A Bacillus subtilis sensor kinase involved in triggering biofilm formation on the roots of tomato plants**. *Molecular microbiology* 2012, **85**(3):418–430-418–430.

4. Burbulys D, Trach KA, Hoch JA: **Initiation of sporulation in B. subtilis is controlled by a multicomponent phosphorelay**. *Cell* 1991, **64**(3):545-552.

5. Kearns DB, Chu F, Branda SS, Kolter R, Losick R: **A master regulator for biofilm formation by Bacillus subtilis**. *Molecular microbiology* 2005, **55**(3):739–749-739–749.

6. Bai U, Mandic-Mulec I, Smith I: **SinI modulates the activity of SinR, a developmental switch protein of Bacillus subtilis, by protein-protein interaction**. *Genes & development* 1993, **7**(1):139-148.

7. Winkelman JT, Blair KM, Kearns DB: **RemA (YlzA) and RemB (YaaB) regulate extracellular matrix operon expression and biofilm formation in Bacillus subtilis**. *Journal of bacteriology* 2009, **191**(12):3981-3991.

8. Nicolas P, Mäder U, Dervyn E, Rochat T, Leduc A, Pigeonneau N, Bidnenko E, Marchadier E, Hoebeke M, Aymerich S: **Condition-dependent transcriptome reveals high-level regulatory architecture in Bacillus subtilis**. *Science* 2012, **335**(6072):1103-1106.

9. El Andari J, Altegoer F, Bange G, Graumann PL: **Bacillus subtilis bactofilins are essential for flagellar hook-and filament assembly and dynamically localize into structures of less than 100 nm diameter underneath the cell membrane**. *PloS one* 2015, **10**(10):e0141546-e0141546.
